# Food and bile micelle binding of quaternary ammonium compounds

**DOI:** 10.5599/admet.2023

**Published:** 2023-09-15

**Authors:** Takeru Sumiji, Kiyohiko Sugano

**Affiliations:** Molecular Pharmaceutics Lab., College of Pharmaceutical Sciences, Ritsumeikan University, 1-1-1, Noji-higashi, Kusatsu, Shiga 525-8577, Japan

**Keywords:** negative food effect, unbound fraction, simulated intestinal fluid, dynamic dialysis, intestinal membrane permeation

## Abstract

**Background and Purpose:**

Physiologically-based biopharmaceutics modeling (PBBM) has been widely used to predict the oral absorption of drugs. However, the prediction of food effects on oral drug absorption is still challenging, especially for negative food effects. Marked negative food effects have been reported in most cases of quaternary ammonium compounds (QAC). However, the mechanism has remained unclear. The purpose of the present study was to investigate the bile micelle and food binding of QACs as a mechanism of the negative food effect.

**Experimental Approach:**

Trospium (TRS), propantheline (PPT), and ambenonium (AMB) were selected as model QAC drugs. The oral absorption of these QACs has been reported to be reduced by 77% (TRS), > 66% (PPT), and 79% (AMB), when taken with food. The fasted and fed state simulated intestinal fluids (FaSSIF and FeSSIF, containing 3 and 15 mM taurocholic acid, respectively) with or without FDA breakfast homogenate (BFH) were used as the simulated intestinal fluid. The unbound fraction (f_u_) of the QACs in these media was measured by dynamic dialysis.

**Key Results:**

The f_u_ ratios (FeSSIF/ FaSSIF) were 0.67 (TRS), 0.47 (PPT), and 0.76 (AMB). When BFH was added to FeSSIF, it was reduced to 0.39 (TRS), 0.28 (PPT), and 0.59 (AMB).

**Conclusion:**

These results suggested that bile micelle and food binding play an important role in the negative food effect on the oral absorption of QACs.

## Introduction

Physiologically-based biopharmaceutics modeling (PBBM) has been widely used to predict the oral absorption of drugs [1]. Food effect prediction is one of the main areas where PBBM is anticipated to be useful [2-8]. However, accurate food effect prediction is still challenging [9-11]. In the fed state, the bile micelle concentration is significantly increased [12]. In addition, various food components and their digestants exist in the gastrointestinal (GI) tract in the fed state. Therefore, the interactions between a drug molecule and bile micelles/ food components can affect oral drug absorption.

A negative food effect is often observed for high solubility/ low permeability drugs (biopharmaceutical classification system (BCS) class III) [13] such as hydrophilic tertiary amines and quaternary ammonium compounds (QAC) [14]. A marked negative food effect has been observed especially in QACs [3]. In most BCS III drugs, the rate and extent of fraction dose absorbed (Fa) is limited by epithelial membrane permeation (Fa rate-limiting step (FaRLS): PL-E) [2,15]. Recently, bile micelle binding was reported to be able to quantitatively elucidate the negative food effect for hydrophilic tertiary amines [3]. On the other hand, for QACs, bile micelle binding alone was not sufficient to elucidate the marked negative food effect [3]. In addition to bile micelles, food components coexist in the fed-state intestine. However, the food binding of QACs has not been investigated.

In PBBM [1,2], the permeation flux (*J*) (dimension: amount/ length^2^/ time) is nominally expressed by the total (= unbound + bound) drug concentration dissolved in the GI fluid (*C*_dissolv_) (dimension: amount/length^3^) and the effective (apparent) permeation coefficient (*P*_eff_) (dimension: length/time) as:


(1)

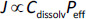



In other words, *P*_eff_ is defined based on the total drug concentration but not the unbound drug concentration (like plasma clearance is based on the plasma concentration in pharmacokinetics). This definition of *P*_eff_ has been employed in PBBM (including commercial software). In the case of PL-E, the effect of the unstirred water layer (UWL) adjacent to the membrane is negligible. Considering that only unbound molecules can permeate the epithelial membrane (the free fraction theory),


(2)

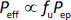



where *f*_u_ is the unbound (free) fraction and *P*_ep_ is the epithelial membrane permeation coefficient of *unbound* molecules (not affected by bile micelle binding). Bile micelle binding reduces the concentration of unbound drug molecules (= *f*_u_ *C*_dissolv_) at the surface of the epithelial membrane, leading to a lower flux. This is expressed as a decrease in *f*_u_ and *P*_eff_ in PBBM. The reduction of *P*_eff_ by bile micelle binding has been reported in the literature [16-18]. The fed/fasted Fa ratio can be approximated as the ratio of the *f*_u_ values in the fasted and fed states (*f_u_*, _fed_ / *f_u_*, _fasted_) for *F*a < 0.7 cases [2].

**Figure 1.**
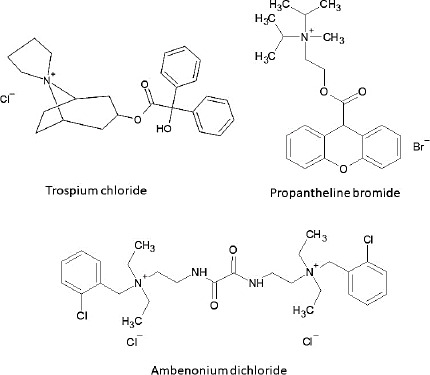
Chemical structures of model QAC drugs

The purpose of the present study was to investigate the bile micelle and food binding of QACs as a mechanism of the negative food effect. Trospium (TRS), propantheline (PPT), and ambenonium (AMB) were selected as model drugs (Figure 1).

The solubility values of these drugs are high (> 100 mg mL^-1^) [3]. QACs are permanently positively charged, showing low organic solvent partition [19] and low artificial membrane permeation [20,21]. The Caco-2 permeability was reported to be 0.20 and 0.15×10^-6^ cm s^-1^ for TRS and AMB, respectively [3]. In fasted-state humans, the Fa values were reported to be about 13 % (TRS) and 6 % (PPT) [22-24]. Therefore, these QACs are classified as high solubility/ low permeability drugs (BCS III), and their oral absorption is limited by epithelial membrane permeation (FaRLS: PL-E). Following postprandial administration, the oral absorption of these QAC drugs is markedly reduced by 77 % (TRS), > 66 % (PPT), and 79 % (AMB), compared to those in the fasted state [25-27]. In this study, the unbound fractions (*f_u_*) of the QACs were measured by dynamic dialysis in the simulated intestinal fluids consisting of bile micelles and food homogenates [28-30]. In a bile micelle solution, bile acids exist as an equilibrium between the monomer and micellar states. When using equilibrium dialysis, bile micelle monomers can diffuse through the dialysis membrane pore and form micelles on the opposite side after equilibrium. Therefore, instead of equilibrium dialysis, dynamic dialysis has been used to determine the micelle binding of drugs.

## Experimental

### Material

Trospium chloride and caffeine were purchased from Tokyo Chemical Industry Co., Ltd (Tokyo, Japan). Sodium chloride (NaCl), sodium dihydrogen phosphate dihydrate (NaH_2_PO_4_ 2H_2_O), 8N NaOH, taurocholic acid (TC), and oleic acid (OA) were purchased from FUJIFILM Wako Pure Chemical Corporation (Osaka, Japan). Propantheline bromide was purchased from Funakoshi Co., Ltd (Tokyo, Japan). Ambenonium dichloride was purchased from Toronto Research Chemicals (Ontario, Canada). Egg yolk lecithin (EL) was purchased from NOF Corporation (Tokyo, Japan). Glyceryl mono-oleate (GM) was purchased from Nippon Surfactant Industries Co., Ltd (Tokyo, Japan). A cellulose dialysis membrane was purchased from As-One Corporation (Osaka, Japan).

The Food and Drug Adminstration (FDA) breakfast ingredients were purchased from the local market (bacon (Itoham Foods Inc, Japan), toast (Choujuku bread, Pasco Shikishima Corporation, Japan), egg (Odama mix, I.T.S Farm Co., Ltd, Japan), hash browns (Hoshino potato, Heinz Japan Ltd, Japan), whole milk (Oishii Megumilk Snow Brand Milk, MEGMILK SNOW BRAND Co., Ltd, Japan), and butter (Hokkaido-butter, MEGMILK SNOW BRAND Co., Ltd, Japan)).

### Methods

#### Processing of FDA Breakfast

The FDA breakfast consisted of one strip of bacon, half a slice of toast, one fried egg, 55 g of hash browns, 30 g of butter, and 100 mL of whole milk. After cooking, the FDA breakfast was homogenized for 15 seconds by a food processor. Before use, the FDA breakfast homogenate (BFH) was divided into small aliquots and stored in a freezer (-30 °C).

#### Measurement of the unbound fraction by dynamic dialysis

Dynamic dialysis was performed by using a side-by-side chamber (SANPLATEC Co., Ltd (Osaka, Japan)) with a cellulose dialysis membrane (molecular weight cut-off: 3500). The membrane area was 2.0 cm^2^. The fluid volume was 1.5 mL on both the donor and acceptor sides. The fasted and fed state simulated intestinal fluids (FaSSIF and FeSSIF, respectively) with or without BFH were used as test media (Table 1) [31]. A test medium containing 0.5 mM of each QAC was added to the donor side and blank FaSSIF was added to the acceptor side. After incubation at 37 °C for 1 h, the concentration of the QAC in the acceptor side was determined by HPLC (Shimazu Prominence LC-20 series, column: Zorbax Eclipse Plus (C18 2.1×50 mm, 3.5 μm) (Agilent Technologies), flow rate: 0.6 mL min^-1^, mobile phase: 0.1 % trifluoroacetic acid acetonitrile/ /water (25 % (TRS), 35 % (PPT), 20 % (AMB), and 10 % (caffeine)), detection: UV (260 nm (TRS), 280 nm (PPT), 265 nm (AMB), and 275 nm (caffeine)), column temperature: 40 °C, and injection volume of 10 μL).

Permeated% was calculated as the ratio of the concentration in the acceptor chamber at 1 h and the theoretical equilibrium concentration (0.25 mM) (× 100 to convert to %). The unbound fraction (*f_u_*) was calculated as the ratio of permeated% in each medium and blank FaSSIF.

## Results

**Figure 2.**
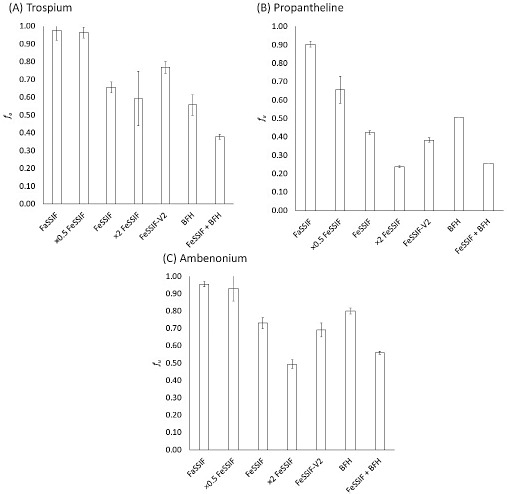
Effect of bile micelles and FDA breakfast homogenate (BFH) on the f_u_ values of QACs. (A) Trospium, (B) propantheline, and (C) ambenonium.

Figure 2 shows the *f*_u_ values of each QAC. Permeated% at 1h, *f*_u_, and the fed/fasted *f*_u_ ratio are summarized in Table 2. The permeated% and the *f*_u_ value of QACs were decreased by bile micelles and BFH, and more significantly decreased when bile micelles and BFH coexisted (decrease by 44 to 74 %). Caffeine was used as a reference un-ionized drug. Permeated% was slightly decreased (by 16 %) in the FeSSIF+BFH condition.

## Discussion

In this study, the bile micelles decreased the *f*_u_ values of the QACs in good agreement with the previous study using the Caco-2 membrane [3]. The addition of BFH to FeSSIF resulted in a more significant decrease in the f_u_ values, comparable to the clinically observed negative food effects on the oral absorption of trospium and propantheline (approximately 70 % reduction). This result suggests that the reduction of *f*_u_ in the fed state is likely to be at least one of the main reasons for the negative food effect on the oral absorption of QACs.

The addition of BFH increases the viscosity of the simulated intestinal fluid. The fluid viscosity could affect the results of dynamic dialysis. To investigate this point, caffeine was used as a control compound. Caffeine is a hydrophilic, non-ionized drug that is unlikely to bind to bile micelles or food components. Therefore, permeated% of caffeine would be mainly affected by fluid viscosity. The permeated% of caffeine in FeSSIF + BFH was reduced markedly less than that of QACs. Therefore, the permeated% of the QACs was mainly affected by the food/bile micelle binding, rather than an increase in fluid viscosity.

BFH contains various components, such as lipids and organic acids. QAC is a cationic compound with lipophilic hydrocarbon moieties (Figure 1). Therefore, both electrostatic and hydrophobic interactions may be formed between a QAC and the components in the FeSSIF+BFH medium. We also investigated the effect of crude bile (ox-bile) on the *f*_u_ value, however, little or no difference was observed between ox-bile and TC (data not shown). Further investigation is required to clarify molecular-level interactions between bile micelles/food and QAC.

Previously, the negative food effect on the oral absorption of trospium has been extensively investigated [25]. The disintegration time and dissolution rate of trospium formulations were reduced due to the increase in fluid viscosity caused by food. It was suggested that this was one of the reasons for the negative food effect [32]. Because trospium is mainly absorbed from the upper small intestine [33], a slowed disintegration time would reduce its absorption [34]. However, a marked negative food effect was also observed when trospium was administered as an oral solution [25]. In addition, if an increase in fluid viscosity were the mechanism for the marked negative food effect, it would have been observed in a wide range of drug products. However, this is not the case. Therefore, an increase in fluid viscosity may not be the main reason for the marked negative food effect on the oral absorption of trospium. In another report, the reduction of ion-pair transport of trospium with a bile acid anion was also suggested as the mechanism of the negative food effect [35]. In that report, it was suggested that bile acids increased the permeation of trospium by ion-pair transport in the fasted state. In the presence of dietary lipids in the fed state, the bile micelle concentration available for ion-pair transport was suggested to be reduced because bile acids are consumed for lipid solubilization. However, an increase in trospium permeability by bile micelles was not statistically significant in Caco-2 [35] and was slightly observed only after 60 min incubation in the rat small intestine [35]. Similarly, in our previous study using Caco-2 cells, bile micelles did not increase the apparent permeability of trospium [3]. Taken together, the results of the present study suggested that the reduction of the unbound fraction by food/bile micelle binding is more likely to be the reason for the negative food effect observed for trospium. This mechanism can also explain the reason that trospium is absorbed from two intestinal absorption windows [25]. A small portion of trospium is first absorbed before binding to food/bile micelles. Food/bile micelle binding would then occur and reduce the membrane flux of trospium. Over time, the food components are digested and absorbed along the GI tract. In addition, bile acids are re-absorbed in the lower part of the small intestine by the site-specific bile acid transporter. Therefore, trospium is released as it transits along the GI tract, resulting in bimodal plasma-concentration time profiles [16,36].

Together with the previous studies [2,3], this study suggested that the *f*_u_ values in the fasted and fed states are required for PBBM to predict the negative food effect. The FeSSIF+BFH medium can be a useful simulated medium to measure the *f*_u_ value *in vitro*. It should be noted that even for high permeability/low solubility drugs (BCS II), the *P*_eff_ values differ between fasted and fed conditions. BCS II roughly corresponds to solubility- UWL permeation limited absorption (FaRLS: SL-U)). Bile micelle binding reduces the effective diffusion coefficient of a drug in the UWL adjacent to the intestinal wall [2,8]. Therefore, the *f_u_* values in the fasted and fed states are required for the quantitative food effects prediction for SL-U (in this case, an increase in *C*_dissolv_ by bile micelles is slightly canceled out by a reduction in *P*_eff_, resulting in a positive food effect [2]). Furthermore, the *f*_u_ value is critical to elucidate the solubility-permeability trade-off for low solubility/low permeability drugs (BCS IV, roughly corresponds to solubility- epithelial membrane permeation limited absorption (FaRLS: SL-E)) [2]. A simple empirical extrapolation from apparent Caco-2 permeability data (*P*_app_) like log *P*_eff_ = *A* log *P*_app_ + *B* should not be used for the food effect prediction by PBBM because the effect of food/ bile micelle binding on *P*_eff_ cannot be considered in this method [37].

For PBBM to be used to waive a clinical food effect study, accurate food effect prediction is required. For diagnosing the bioequivalence between the fasted and fed states, the prediction error must be less than 20 %. To achieve this goal, further investigation on food and bile micelle binding is required. Food digestion would change the chemical composition of the digest. The effect of food digestion on the *f*_u_ value would be of great interest and should be investigated in the future.

The *f*_u_ measurement of high solubility/low permeability drugs has been challenging. The Caco-2 assay has previously been used for the *f*_u_ measurement [3]. However, Caco-2 cells may be damaged by food components. In the case of transcellular permeation, parallel artificial membrane permeation assay (PAMPA) may be used to assess the food and bile micelle binding of a drug [38,39]. However, because QACs are permanently charged, they are absorbed mainly via the paracellular route [40]. In such cases, dynamic dialysis would be a good tool to assess the food and bile micelle binding of a drug.

## Conclusions

In conclusion, the results of this study suggested that the reduction of *f*_u_ in the fed state is likely to be the reason for the negative food effect on the oral absorption of QACs. The FeSSIF+BFH medium can be useful for assessing the food and bile micelle binding of a drug. Dynamic dialysis would be a good tool to measure a *f*_u_ value in a medium containing bile micelles.

## Figures and Tables

**Table 1.** Composition of test media^*^

Test medium	TC, mM	EL, mM	GM, mM	OA, mM	BFH, wt.%
FaSSIF	3	0.75	0	0	0
×0.5 FeSSIF	7.5	1.88	0	0	0
FeSSIF	15	3.75	0	0	0
×2FeSSIF	30	7.50	0	0	0
FeSSIFv2	10	2.0	5	0.8	0
BFH added to blank FaSSIF	0	0	0	0	50
FeSSIF+BFH	15	3.75	0	0	50

^*^The components were dissolved in a phosphate buffer (phosphate 28.6 mM, NaCl 106 mM, pH 6.5; blank FaSSIF); QAC: 0.5 mM

**Table 2.** Permeated% and *f_u_*

Drug	Test medium	Permeated% at 1 h^*^	*f*_u_^*^	*f*_u_ ratio^*,**^
Trospium	Blank FaSSIF	21.9 ± 0.1	-	-
	FaSSIF	21.4 ± 1.0	0.98 ± 0.06	-
	×0.5 FeSSIF	21.1 ± 0.5	0.96 ± 0.03	0.99 ± 0.07
	FeSSIF	14.4 ± 0.6	0.66 ± 0.03	0.67 ± 0.02
	×2 FeSSIF	13.0 ± 2.7	0.59 ± 0.15	0.61 ± 0.14
	FeSSIF-V2	16.8 ± 0.6	0.77 ± 0.03	0.79 ± 0.01
	BFH	12.2 ± 1.0	0.56 ± 0.06	0.57 ± 0.03
	FeSSIF + BFH	8.3 ± 0.3	0.38 ± 0.02	0.39 ± 0.02
Propantheline	Blank FaSSIF	25.6 ± 0.2	-	-
	FaSSIF	23.2 ± 0.3	0.90 ± 0.02	-
	×0.5 FeSSIF	16.8 ± 1.5	0.66 ± 0.07	0.73 ± 0.08
	FeSSIF	10.9 ± 0.2	0.42 ± 0.01	0.47 ± 0.02
	×2 FeSSIF	6.1 ± 0.1	0.24 ± 0.01	0.26 ± 0.01
	FeSSIF-V2	9.8 ± 0.3	0.38 ± 0.01	0.42 ± 0.01
	BFH	13.0 ± 1.0	0.51 ± 0.00	0.56 ± 0.05
	FeSSIF + BFH	6.6 ± 0.6	0.26 ± 0.00	0.28 ± 0.03
Ambenonium	Blank FaSSIF	17.5 ± 1.0	-	-
	FaSSIF	16.8 ± 0.2	0.96 ± 0.02	-
	×0.5 FeSSIF	16.3 ± 1.0	0.93 ± 0.07	0.97 ± 0.06
	FeSSIF	12.8 ± 0.4	0.73 ± 0.03	0.76 ± 0.02
	×2 FeSSIF	8.7 ± 0.4	0.49 ± 0.03	0.52 ± 0.03
	FeSSIF-V2	12.1 ± 0.6	0.69 ± 0.04	0.72 ± 0.03
	BFH	14.0 ± 0.3	0.80 ± 0.02	0.84 ± 0.01
	FeSSIF + BFH	9.8 ± 0.1	0.56 ± 0.01	0.59 ± 0.02
Caffeine	Blank FaSSIF	40.1 ± 0.7		-
	BFH	36.4 ± 2.8	0.91 ± 0.09	-
	FeSSIF + BFH	33.9 ± 0.8	0.84 ± 0.03	-

^*^mean ± S.D., *n* = 3

^**^vs. the *f_u_* value in FaSSIF
